# The identification of 14 new genes for meat quality traits in chicken using a genome-wide association study

**DOI:** 10.1186/1471-2164-14-458

**Published:** 2013-07-08

**Authors:** Yanfa Sun, Guiping Zhao, Ranran Liu, Maiqing Zheng, Yaodong Hu, Dan Wu, Lei Zhang, Peng Li, Jie Wen

**Affiliations:** 1 Institute of Animal Science, Chinese Academy of Agricultural Sciences, Beijing 100193, P.R. China; 2 State Key Laboratory of Animal Nutrition, Beijing 100193, P.R. China; 3 College of Animal Science and Technology, Yangzhou University, YangzhouJiangsu 225009, P.R. China

**Keywords:** Chicken, GWAS, Meat quality traits, Abdominal fat, Candidate genes

## Abstract

**Background:**

Meat quality is an important economic trait in chickens. To identify loci and genes associated with meat quality traits, we conducted a genome-wide association study (GWAS) of F2 populations derived from a local Chinese breed (Beijing-You chickens) and a commercial fast-growing broiler line (Cobb-Vantress).

**Results:**

In the present study, 33 association signals were detected from the compressed mixed linear model (MLM) for 10 meat quality traits: dry matter in breast muscle (DM_Br_), dry matter in thigh muscle (DM_Th_), intramuscular fat content in breast muscle (IMF_Br_), meat color lightness (L*) and yellowness (b*) values, skin color L*, a* (redness) and b* values, abdominal fat weight (AbFW) and AbFW as a percentage of eviscerated weight (AbFP). Relative expressions of candidate genes identified near significant signals were compared using samples of chickens with High and Low phenotypic values. A total of 14 genes associated with IMF_Br_, meat color L*, AbFW, and AbFP, were differentially expressed between the High and Low phenotypic groups. These genes are, therefore, prospective candidate genes for meat quality traits: *protein tyrosine kinase* (*TYRO3*) and *microsomal glutathione S-transferase 1* (*MGST1*) for IMF_Br_; *collagen, type I, alpha 2* (*COL1A2*) for meat color L*; and *RET proto-oncogene* (*RET*), *natriuretic peptide B* (*NPPB*) and *sterol regulatory element binding transcription factor 1* (*SREBF1*) for the abdominal fat (AbF) traits.

**Conclusions:**

Based on the association signals and differential expression of nearby genes, 14 candidate loci and genes for IMF_Br_, meat L* and b* values, and AbF are identified. The results provide new insight into the molecular mechanisms underlying meat quality traits in chickens.

## Background

Meat quality in chickens is an important trait and includes pH, meat color, drip loss, tenderness, intramuscular fat (IMF) content, and other fat traits such as the contents and proportions of abdominal and subcutaneous fat. The selection of broiler chickens, initially focused on increasing growth performance and improving body composition [1], also led to indirect and often deleterious effects on meat quality traits, particularly excessive deposition of abdominal fat (AbF), the formation of which represents inefficient use of feed [2,3]. The elucidation of the molecular mechanisms underlying meat quality traits in chickens will have both biological and economic consequences.

Quantitative trait loci (QTLs) for many traits in chicken have been studied for over 20 years; 52 QTLs for meat-quality traits and 272 for abdominal fat traits have been detected in a variety of chicken chromosomal regions [4]. These QTLs were detected by linkage analysis and by candidate gene analysis. Both of these methods have limitations: the identified QTL regions are generally large and require subsequent fine mapping to identify closely linked markers or causative variants. Candidate genes, based on putative physiological roles, may exclude the identification of novel genes or pathways that influence the target traits [5].

The currently available chicken 60 K SNP chip covers the entire genome [5,6]. Genome-wide association studies (GWAS) can aid in more precisely identifying the genes and variants underlying important traits. In chicken, GWAS have already been performed for growth [7,8], egg production and quality [9] and disease resistance [10]. In the present study, we have performed a GWAS of several meat-quality traits in an F2 resource population derived from a cross between a Chinese local breed (Beijing-You, highly regarded for its meat quality) and a commercial rapidly-growing broiler line (Cobb-Vantress) to identify candidate genes.

## Results

### Phenotype statistics

The descriptive statistics for 16 meat quality traits in the F2 resource population used for the present GWAS are shown in Table 1. All non-normal phenotypic data, intramuscular fat content in thigh muscle (IMF_Th_), drip loss (DL), meat redness value (a*) and yellowness value (b*) of breast muscle, shear force (SF) of the pectoral major muscle, skin a* and b*, were normalised by Box-Cox or Johnson transformation except those for abdominal fat weight (AbFW), percentage of AbFW to eviscerated weight (AbFP) and the ultimate pH (24 h) of breast muscle (pHu).

**Table 1:** Descriptive statistics for the meat quality traits

**Traits (Unit)**^**1**^	**N**	**Mean**	**SD**	**Min**	**Max**	**CV**
DM_Br_ (%)	316	27.87	1.30	20.97	33.29	4.67
DM_Th_ (%)	309	24.39	1.14	20.47	29.62	4.69
IMF_Br_ (%)	316	2.73	0.97	0.48	6.04	35.67
IMF_Th_ (%)	310	7.17	2.23	1.39	15.24	31.10
AbFW (g)	324	37.47	29.60	0.00	128.30	88.43
AbFP (%)	324	1.43	1.21	0.00	5.64	84.61
SFT (mm)	323	5.22	1.82	1.16	11.36	34.75
pHu	304	5.55	0.25	5.00	6.30	4.43
DL (%)	315	7.03	2.94	1.66	18.67	41.78
SF (kg/cm^2^)	311	3.77	0.95	2.04	6.45	0.03
Meat color						
L*	293	57.62	3.47	48.61	66.48	6.02
a*	292	12.18	2.12	7.37	19.45	17.44
b*	294	15.85	3.39	8.18	25.31	21.42
Skin color						
L*	270	65.33	4.79	50.72	75.90	7.34
a*	266	8.61	3.40	1.80	17.24	39.55
b*	267	11.03	4.53	1.12	24.98	41.04

^1^*DM*_*Br*_ dry matter content in breast muscle, *DM*_*Th*_ dry matter content in thigh muscle, *IMF*_*Br*_ intramuscular fat content in breast muscle, *IMF*_*Th*_ intramuscular fat content in thigh muscle, *AbFW* abdominal fat weight, *AbFP* percentage of AbFW to eviscerated weight, *SFT* subcutaneous fat thickness, *pHu* the ultimate pH (24 h) of breast muscle, *DL* drip loss, *SF* shear force of the pectoral major muscle, L* lightness value; a* redness value and b* yellowness value. The same abbreviations are used in the following table.

### GWAS analysis

**Figure 1:**
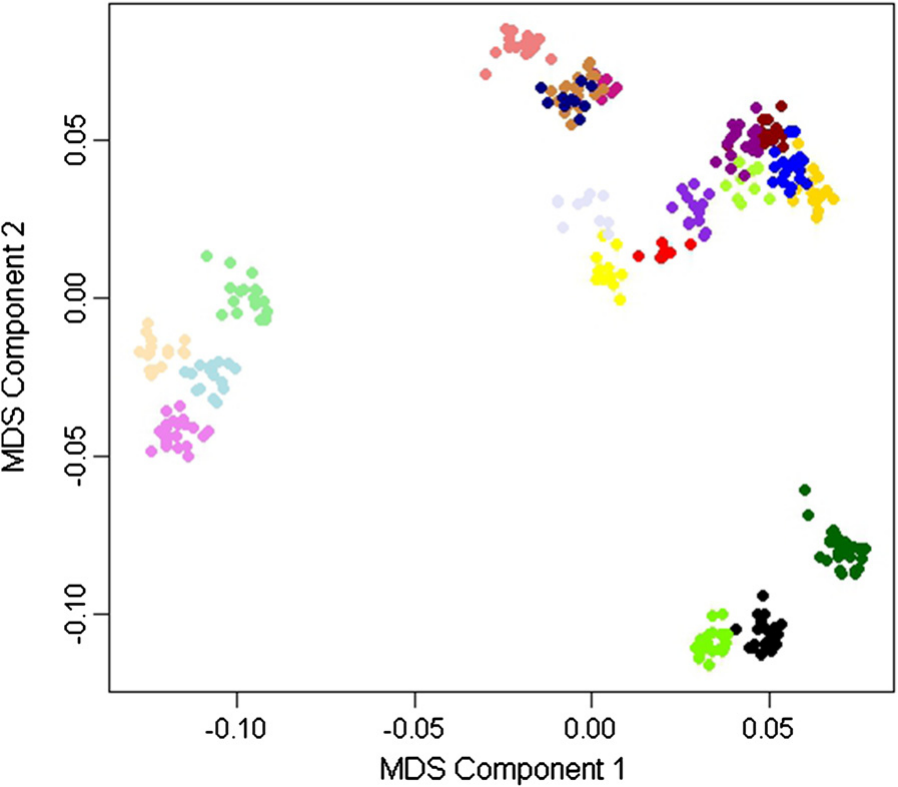
**Population structure identification with multidimensional scaling analysis.** Full-sib families are shown in the same color.

A total of 6,695 independent SNP markers, distributed on all autosomes, were obtained with r^2^ = 0.2 Multidimensional scaling (MDS) analysis of these SNPs using the first two principal components (Figure 1) indicated that chickens within each full-sib family were clustered together. To correct for population stratification, the first MDS component was used as a covariate in a general linear model (GLM) and a compressed mixed linear model (MLM), as suggested in previous studies [8,11]. The relative kinship matrix was constructed from these independent SNP markers as a random effect in the compressed MLM.

**Figure 2:**
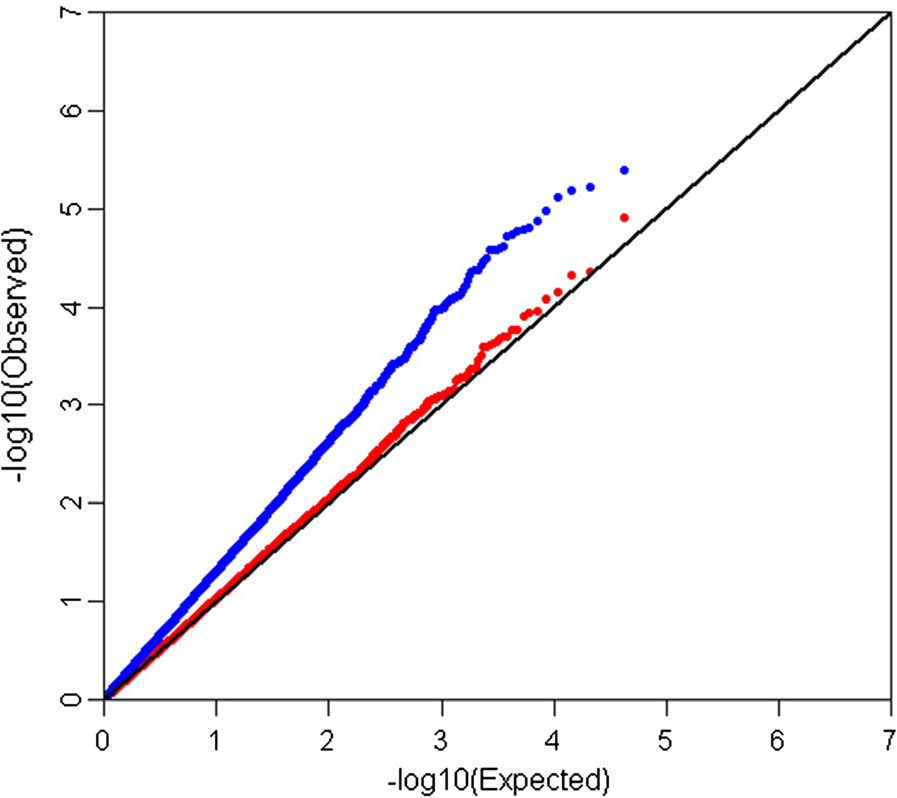
Quantile-quantile plot of the general linear model (GLM) (blue) and compressed mixed linear model (MLM) (red) for abdominal fat weight (AbFW).

**Figure 3:**
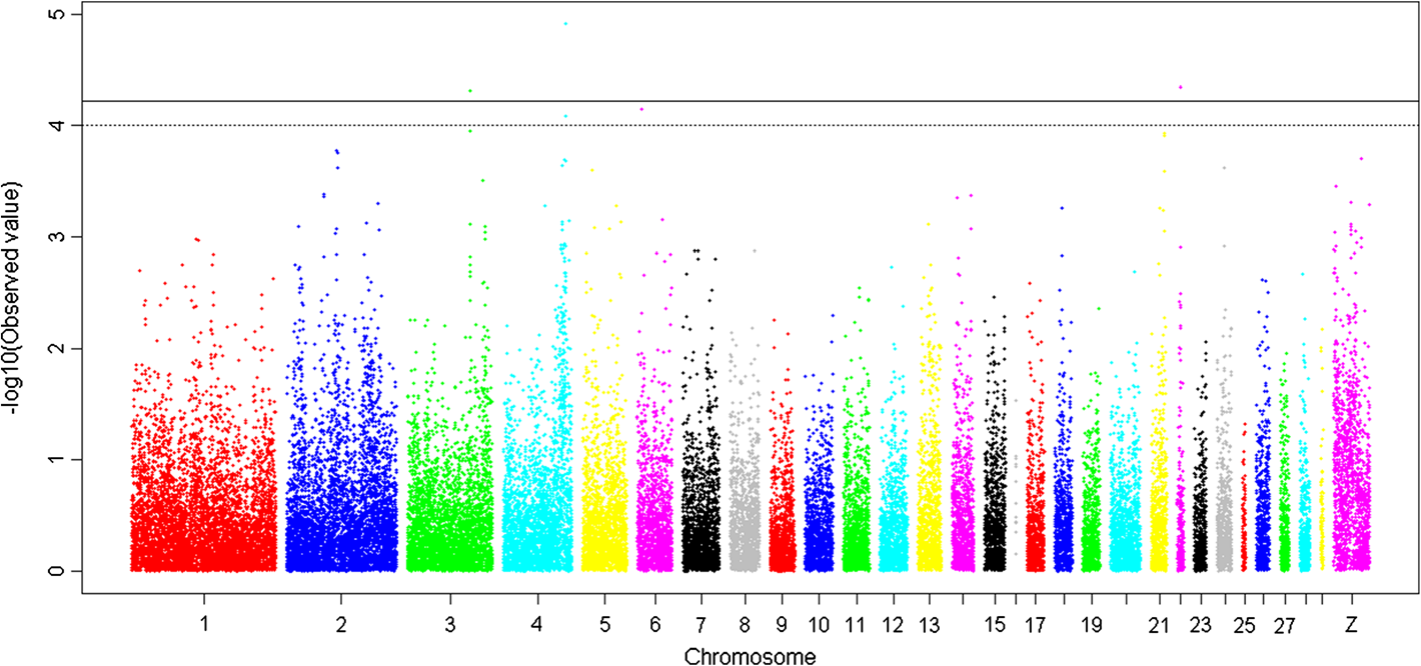
**A Manhattan plot showing the association of all SNPs with abdominal fat weight (AbFW) from the compressed mixed linear model (MLM).** SNPs are plotted on the x-axis according to their position on each chromosome against their association with AbFW on the y-axis (as -log10 p-value). The dashed line indicates genome-wide association (p = 1.00 × 10^-4^), and the solid line indicates significance with a p-value threshold of 5.96 × 10^-5^.

**Figure 4:**
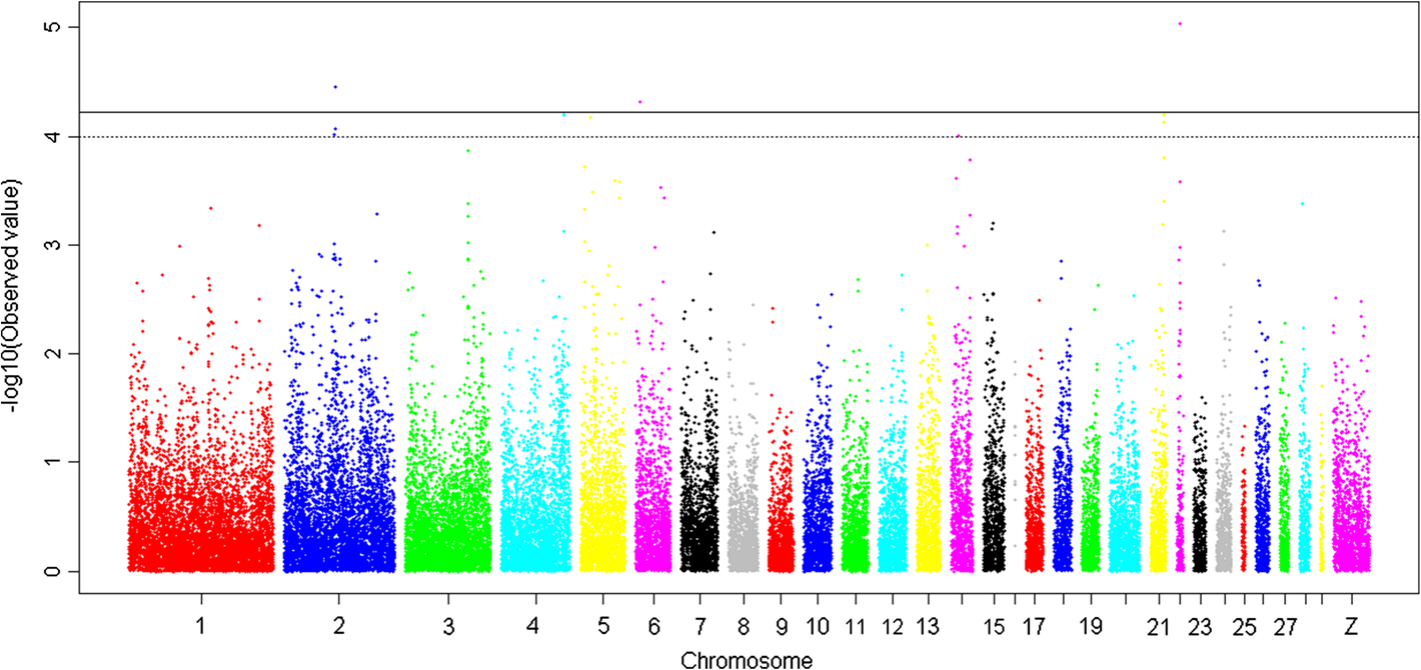
**A Manhattan plot showing the association of all SNPs with percentage of abdominal fat (AbFP).** The data and form of presentation are exactly as described for Figure 3 except the trait is AbFP, the proportion of eviscerated weight represented by abdominal fat.

As observed in Figure 2, the compressed MLM is more effective than the GLM for controlling population structure, as described in previous studies [12-17]. The compressed MLM was, therefore, the preferred model to identify association signals. In addition, the compressed MLM also increases false negatives while false positives are reduced [13-16]. The suggestive significance threshold for p-value was set at 1.0 × 10^-4^ in the MLM analyses and those reaching genome-wide significance (p < 2.98 × 10^-6^) from the GLM analysis are also indicated. The 33 SNPs with association signals (p < 1.0 × 10^-4^) are listed in Tables 2 and 3 and Manhattan plots are shown in Figures 3 and 4 and Additional file 1: Figure S1. Seven of the 33 reached genome-wide significance based on the GLM analysis.

**Table 2:** **Location of and gene information for SNPs associated (P < 1 × 10**^**-4**^**) with meat quality traits**

**Trait**	**Chromosome**	**SNPs**	**Position**	**Alleles**	**MAF**^**1**^	**P_value**^**2**^	**P_adjust**^**3**^	**R**^**2 4**^	**Nearest Gene**	**Distance (Kb)**^**5**^
DM_Br_	2	Gga_rs14206277	78152161	G/A	0.338	2.01E-05	0.34	0.071	*FAM105A*	within
DM_Th_	14	Gga_rs14086206	14720651	A/G	0.340	8.38E-05	1.40	0.077	*TBC1D24*	D 20.6
DM_Br_	Z	GGaluGA346234	1626487	G/A	0.016	1.16E-05	0.19	0.075	*ST8SIA5*	within
IMF_Br_	1	Gga_rs13878108	65617257	G/A	0.240	6.49E-05	1.09	0.063	*MGST1*	D 183.1
IMF_Br_	3	GGaluGA217414	40654853	A/G	0.116	9.30E-05/**2.59E-06**	1.56	0.061	*NTPCR*	U 109.9
IMF_Br_	4	GGaluGA255658	43311293	G/A	0.100	3.46E-05/**5.61E-09**	0.58	0.057	*AGA*	U 330.8
IMF_Br_	5	Gga_rs14524377	27009717	G/A	0.193	5.41E-05	0.91	0.066	*TYRO3*	U 73.6
IMF_Br_	Z	GGaluGA348872	18701354	A/G	0.025	2.14E-05/**4.28E-07**	0.36	0.059	*KIF2A*	U 122.3
Meat color										
L*	2	GGaluGA137635	23472767	A/G	0.302	7.04E-05	1.18	0.068	*COL1A2*	U 58.0
L*	18	GGaluGA121429	6990031	C/A	0.412	8.96E-05	1.50	0.065	*KPNA2*	U 0.2
L*	18	GGaluGA121456	7112093	G/A	0.383	7.19E-05	1.21	0.068	*PSMD12*	U 34.3
b*	27	Gga_rs15237721	1597620	G/A	0.364	4.85E-05	0.81	0.077	*FTSJ3*	U 2.4
Skin color										
L*	2	GGaluGA159190	99182734	G/A	0.122	7.62E-05	1.28	0.073	*PTPN2*	U 130.6
L*	13	Gga_rs14058862	10586210	A/G	0.114	9.39E-05	1.57	0.071	*SPINK5*	U 28.1
L*	13	Gga_rs15697794	11284741	G/A	0.049	9.61E-05	1.61	0.058	*CLINT1*	U 15.6
L*	21	Gga_rs16180462	3645146	G/A	0.162	8.76E-05	1.47	0.072	*PGD*	U 62.7
a*	19	Gga_rs15044922	2083895	G/A	0.213	5.10E-05	0.85	0.077	*MIR1587*	D 301.0
b*	6	GGaluGA305921	33306370	A/G	0.412	3.24E-05	0.54	0.089	*BUB3*	D 195.5

^1^*MAF* minor allele frequency; ^2^the bold p-values of SNPs are the genome-wide significance by the general linear model (GLM); ^3^p-adjust indicates the p-value adjusted by “LD adjusted” Bonferroni; ^4^R^2^, SNP indicates the ratio of phenotypic variation; ^5^U indicates that the SNP is upstream of the gene, while D indicates that the SNP is downstream of the gene. These abbreviations are also used in Table 3.

**Table 3:** **Location of and gene information for SNPs associated (P < 1 × 10**^**-4**^**) with abdominal fat traits**

**Trait**	**Chromosome**	**SNPs**	**Position**	**Alleles**	**MAF**	**P_value**	**P_adjust**	**R**^**2**^	**Nearest Gene**	**Distance (Kb)**
AbFW	3	Gga_rs14385159	83714232	A/G	0.248	4.79E-05	0.80	0.052	*COL12A1*	D 65.5
AbFW	4	Gga_rs15634423	85973459	A/G	0.203	1.22E-05	0.20	0.059	*MXD4*	D 82.4
AbFW	4	Gga_rs16445604	86019649	A/G	0.239	8.28E-05	1.39	0.049	*MXD4*	D 128.6
AbFW	6	Gga_rs14564481	5917270	A/G	0.121	7.03E-05/**4.11E-07**	1.18	0.050	*RET*	within
AbFW	22	Gga_rs14708241	2145071	G/A	0.104	4.46E-05/**2.34E-07**	0.75	0.052	*LOC431251*	U 124.3
AbFP	2	Gga_rs15112628	68073875	A/G	0.223	9.62E-05	1.61	0.059	*FOXC1*	U 124.7
AbFP	2	Gga_rs13710186	68939093	G/A	0.166	3.53E-05	0.59	0.066	*VPS4B*	U 63.6
AbFP	2	Gga_rs14201030	69308190	G/A	0.290	8.49E-05	1.42	0.060	*BCL2*	D 160.4
AbFP	4	Gga_rs15634423	85973459	A/G	0.203	6.37E-05	1.07	0.062	*MXD4*	D 82.4
AbFP	5	GGaluGA276197	15378139	G/A	0.132	6.59E-05	1.10	0.062	*BRSK2*	U 54.0
AbFP	6	Gga_rs14564481	5917270	A/G	0.121	4.88E-05/**2.62E-08**	0.82	0.064	*RET*	within
AbFP	21	Gga_rs14285437	5764093	A/G	0.247	6.40E-05	1.07	0.062	*NPPB*	within
AbFP	21	Gga_rs14285449	5774061	G/A	0.258	7.30E-05	1.22	0.061	*NPPB*	D 8.3
AbFP	22	Gga_rs14708241	2145071	G/A	0.104	9.23E-06/**6.58E-08**	0.15	0.075	*LOC431251*	U 124.3
AbFP	14	GGaluGA101040	4941190	G/A	0.245	9.94E-05	1.67	0.059	*SREBF1*	D 6.3

### Dry matter content in breast (DM_Br_) and (DM_Th_)

Two SNPs were associated with DM_Br_ (p = 1.16 × 10^-5^ and 2.01 × 10^-5^). They were located at 0.16 Mb on chicken (*Gallus gallus*) chromosome Z (GGZ) and 7.81 Mb on GGA2, within the genes for *ST8 alpha-N-acetyl-neuraminide alpha-2,8-sialyltransferase 5* (*ST8SIA5*) and *family with sequence similarity 105, member A* (*FAM105A*), respectively. One SNP (Gga_rs14086206), located at 1.47 Mb on GGA14, was associated with DM_Th_ (p = 8.38 × 10^-5^) and was located 20.6 Kb downstream from the *TBC1 domain family member 24* gene (*TBC1D24*).

### Intramuscular fat content in breast muscle (IMF_Br_)

Genes related to lipid metabolism would be predicted to influence IMF_Br_. Five SNPs associated with this trait were identified by the compressed MLM (p < 1.0 × 10^-4^), of which three were of genome-wide significance by GLM analysis (p < 2.98 × 10^-6^). One SNP (GGaluGA348872), located at 1.87 Mb on GGZ, had highly significant association with IMF_Br_ (p = 2.14 × 10^-5^) and is located 122.3 Kb upstream of the *kinesin heavy chain member 2A* (*KIF2A*) gene. Another SNP (GGaluGA255658), located at 4.33 Mb on GGA4, was also associated with IMF_Br_ (p = 3.46 × 10^-5^) and is located 330.8 Kb upstream of the *aspartylglucosaminidase* (*AGA*) gene. Three more SNPs (Gga_rs14524377, Gga_rs13878108 and GGaluGA217414) having association with IMF_Br_ were in the proximity of *protein tyrosine kinase* (*TYRO3*), *microsomal glutathione S-transferase 1* (*MGST1*) and *nucleoside-triphosphatase, cancer-related* (*NTPCR*) genes, on GGA5, GGA1 and GGA3, respectively.

### Meat color traits

Three SNPs were associated with meat color lightness (L*) (p < 1.0 × 10^-4^). One SNP, located at 2.35 Mb on GGA2, is 58.0 Kb away from the *collagen, type I, alpha 2* (*COL1A2*) gene. The other two SNPs are located within the 0.12 Mb segment (between 7.11 Mb to 6.99 Mb) on GGA18. These SNPs are approximately 34.3 and 0.20 Kb away from the *proteasome (prosome, macropain) 26S subunit, non-ATPase, 12* (*PSMD12*) *and karyopherin alpha 2 (RAG cohort 1, importin alpha 1)* (*KPNA2*) genes, respectively. The SNP (Gga_rs15237721), located at 0.16 Mb on GGA27, was associated (p = 4.85 × 10^-5^) with meat color b*; the nearest gene, *FtsJ homolog 3* (*E. coli*) (*FTSJ3*) gene, is located 2.4 Kb upstream.

### Skin color traits

Four SNPs were found to be associated with skin color L* (p < 1.0 × 10^-4^). One, located at 9.92 Mb on GGA2, is 130.6 Kb upstream of the *tyrosine-protein phosphatase non-receptor type 2* (*PTPN2*) gene. The SNP identified on GGA21 is 62.7 Kb head of the *phosphogluconate dehydrogenase* (*PGD*) gene. Two SNPs, located within a 0.70 Mb segment (10.59 Mb to 11.28 Mb) on GGA13, are approximately 28.1 and 15.6 Kb ahead of the *serine peptidase inhibitor, Kazal type 5* (*SPINK5*) and *clathrin interactor 1* (*CLINT1*) genes, respectively.

The SNP Gga_rs15044922 on GGA19 was associated with skin a* (p = 5.10 × 10^-5^), and is located distal (301 Kb) to a microRNA cluster that includes three microRNAs (*MIR1587*, *MIR1354* and *MIR1567*). Another SNP, GGaluGA305921, located at 3.33 Mb on GGA6, was associated with skin b* (p = 3.24 × 10^-5^). The known gene nearest to this SNP (195 Kb) is *budding uninhibited by benzimidazoles 3 homolog (yeast)* (*BUB3*).

### Abdominal fat (AbF) traits

Variants at 15 loci were identified as SNPs associated with AbF traits by compressed MLM (p < 1.0 × 10^-4^), and four were of genome-wide significance by GLM (p < 2.98 × 10^-6^) (Table 3, and Figures 3, 4). Two SNPs (Gga_rs15634423 and Gga_rs16445604) located within a 46.19 Kb region on GGA4 were associated with AbFW (p < 1.0 × 10^-4^); these SNPs are both downstream (82.4 Kb and 128.6 Kb) of the *max-interacting transcriptional repressor MAD4* (*MXD4*) gene. The SNP Gga_rs15634423 was also associated with AbFP (p = 6.37 × 10^-5^). Another SNP (Gga_rs14708241), located at 0.21 Mb on GGA22, was associated with both AbFW and AbFP (p = 4.46 × 10^-5^ and p = 9.23 × 10^-6^, respectively). This SNP is 124.3 Kb away from the nearest known gene, *LOC431251*, which encodes *prolactin-releasing peptide receptor-like protein* (PrRPR), also known as *G-protein coupled receptor 10* (GPR10). SNP Gga_rs14385159, located at 8.37 Mb on GGA3, is 65.5 Kb downstream of *Collagen, type XII, alpha 1* (*COL12A1*) and was associated with AbFW (p = 4.79 × 10^-5^). Notably, a SNP (Gga_rs14564481) within the *RET proto-oncogene (RET)* gene on GGA6, was associated with both AbFW and AbFP (p < 1.0 × 10^-4^).

Three SNPs located within a 1.23 Mb region between 68.07 Mb and 69.31 Mb on GGA1 were associated with AbFP (p <1.0 × 10^-4^). One of these SNPS is 63.6 Kb upstream of the *vacuolar protein sorting-associated protein 4B* (*VPS4B*) gene, and another is approximately 160.4 Kb away from the known *B-cell lymphoma 2* (*BCL2*) gene. The third SNP is located 124.7 Kb upstream of the *forkhead box C1* (*FOXC1*) gene. On GGA21, two SNPs were associated with AbFP (p < 1.0 × 10^-4^). These SNPs are within 8.3 Kb of the *natriuretic peptide B* (*NPPB*) gene. Another SNP, located at 54.0 Kb on GGA5, was associated with AbFP and is 54.0 Kb away from the nearest known gene, *BR serine/threonine kinase 2* (*BRSK2*). Interestingly, one SNP located at 0.49 Mb on GGA14 was associated with AbFP and is 6.3 Kb downstream from the gene for *adipocyte determination- and differentiation-dependent factor 1*, the *sterol regulatory element binding transcription factor 1* (*SREBF1*).

No SNPs were found to be associated with IMF_Th_, SFT, pHu, DL, SF and meat color a* of breast muscle.

### The mRNA expression study of candidate genes identified by the GWAS

Based on the GWAS analysis, 17 candidate genes containing or in proximity of SNPs with trait association were further evaluated by real-time quantitative PCR (Q-PCR) in subsets of six chickens with lowest or highest trait phenotypic values (Low, High). Significant differential expression (p < 0.05) between the Low and High birds was demonstrated for 14 of the 17 (Table 4).

**Table 4:** Relative mRNA abundance in the low and high groups

**Traits**	**Genes**	**Groups**^**1**^	
		**Low**	**High**
IMF_Br_	*KIF2A*	1.00 ± 0.05**	0.55 ± 0.23
	*TYRO3*	1.00 ± 0.10**	0.25 ± 0.11
	*MGST1*	1.01 ± 0.13**	0.24 ± 0.04
	*NTPCR*	1.00 ± 0.04**	0.35 ± 0.22
AbFW	*COL12A1*	1.02 ± 0.24*	0.57 ± 0.17
	*RET*	1.02 ± 0.23	31.16 ± 0.68**
AbFP	*VPS4B*	1.03 ± 0.29**	0.48 ± 0.17
	*NPPB*	1.12 ± 0.14	72.08 ± 9.21**
	*BRSK2*	1.01 ± 0.17**	0.10 ± 0.04
	*FOXC1*	1.04 ± 0.33**	0.30 ± 0.12
	*SREBF1*	1.02 ± 0.24**	0.07 ± 0.04
Meat color L*	*COL1A2*	1.01 ± 0.19**	0.24 ± 0.19
	*PSMD12*	1.01 ± 0.16*	0.67 ± 0.04
	*KPNA2*	1.00 ± 0.09	1.76 ± 0.37*

^1^High group (n = 6) consisting of samples from chicken with the highest trait values and Low group (n = 6) with the lowest trait values; * p < 0.05, **p < 0.01.

For IMF_Br_, transcript abundance of four (of the five) genes identified near significant SNPs was significantly down-regulated in the High group compared to the Low group (p < 0.01); the differentially expressed genes were *KIF2A, TYRO3, MGST1* and *NTPCR.*

For meat color L*, three of four chosen genes were differentially expressed between Low and High phenotypic groups (p < 0.05 or 0.01). Transcript abundance of *COL1A2* and *PSMD12* was significantly down-regulated in the High group (p < 0.01, 0.05), and that of *KPNA2* was significantly higher (p < 0.01).

Of the eight genes identified in connection with AbF traits, seven were differentially expressed between the Low and High group (p < 0.01). Compared to the Low group, the expression of *RET* and *NPPB* was significantly increased (p < 0.01), and that of *COL12A1*, *VPS4B*, *BRSK2*, *FOXC1* and *SREBF* was decreased (p < 0.05 or 0.01) in the High group.

## Discussion

### Genome-wide association analysis

There has been effective use of GWAS in meat quality and carcass traits in other species and narrow regions or SNPs associated with pork and beef quality have been revealed [18,19]. Here, we present a GWAS of meat quality traits in an F2 chicken population derived from a cross of Beijing-You chickens and commercial fast-growing broilers.

### Meat quality traits

Dry matter content (DM) is a primary muscle characteristic. Nones *et al*. (2012) mapped a QTL for the water content (100-DM) of a chicken carcass at 23 cM on GGA27 in a half-sib linkage analysis [20]. In the present study, two loci for DM content in breast muscle (DM_Br_) and one for DM_Th_ have been identified. Two SNPs within *ST8SIA5* and *FAM105A* genes were associated with DM_Br_. *ST8SIA5* encodes a type II membrane protein, a member of glycosyltransferase family 29 playing a role in the synthesis of some gangliosides and having a function in cellular recognition and cell-to-cell communication [21]. The pro-apoptotic gene *FAM105A*, when overexpressed, leads to cell apoptosis [22]. A SNP near *TBC1D2* was identified to be associated with DM_Th_. This gene encodes a protein with a conserved domain, the TBC domain, common in proteins interacting with GTPases and has been related to endocytic trafficking [23]. Function characterization of these genes, near SNPs associated with muscle DM in chickens, is not yet clear.

Intramuscular fat (IMF) is an important determinant of meat quality influencing the tenderness, juiciness and flavour of meat [24]. In the present study, four genes (*TYRO3*, *MGST1*, *KIF2A*, and *NTPCR*) are shown to be potentially related to IMF_Br_. These genes were all differentially expressed in chickens with low and high IMF_Br_ (Table 4), and some are known to play roles in lipid metabolism. *TYRO3* plays an important role in cell proliferation and differentiation and has been associated with adipocyte size in moderately obese individuals in a clinical study [25]. The protein encoded by *MGST1* catalyses the conjugation of glutathione to electrophiles and the reduction of lipid hydroperoxides. This gene was differentially expressed in the longissimus dorsi of Northeastern Indigenous and Large White pigs [26] and was previously identified in a QTL for chicken IMF_Br_[27]. *KIF2A* encodes the kinesin-like protein KIF2A, a microtubule-associated motor protein. It can regulate microtubule dynamics at the growth cone edge by depolymerizing microtubules and plays a role in the suppression of collateral branch extension [28]. *NTPCR* is a cancer-related gene with a presumed role in human tumorigenesis [29]. No previous studies have linked *KIF2A* or *NTPCR* with IMF_Br_ and, as the associations were strong (Table 2) and the differential expression was quite large, further study of these genes seems to be warranted.

Meat color is an important quality that influences consumer acceptance of poultry meat and has significant positive correlations with pH and water-holding capacity [30]. Le Bihan-Duval *et al*. (2011) identified an influence of *beta-carotene dioxygenase 1* (*BCDO1*) on chicken breast meat color using classical QTL analysis and gene expression QTL (eQTL) [31]. In the present study, we did not observe an influence of *BCDO2* on meat color, probably because a different chicken population was tested. Three new genes for breast meat color were expressed differentially between the High and Low phenotypic groups. *COL1A2*, which encodes one of the chains of type I collagen, was differentially expressed here (4-fold) and also between the red and white skeletal muscles of Chinese Meishan pigs [32]; this gene could well be a candidate gene for meat color in chickens. *PSMD12*, encoding a proteasomal regulatory subunit, has been associated with liver function in humans [33]. *KPNA2* encodes an importin, functioning in retrograde transport of signaling molecules from the axonal growth cone to the nucleus [34]. How the *PSMD12* and *KPNA2* genes might function in influencing meat color in chickens is not known.

The color of chicken skin influences consumer appeal and hence is also an important phenotype. A previous study found that yellow skin was caused by one or more cis-acting and tissue-specific regulatory mutation(s) that inhibit expression of *BCDO2* in the skin [35]. Six SNPs were identified here as being associated with skin color but the only one associated with yellowness was near the *BUB3* gene, with unknown function in chickens. One of genes with known effects on skin color, close to a SNP of significance, is *SPINK5*. The skin of *SPINK5*-deficient mice has large intensely blue areas close to colorless regions [36].

### Abdominal fat traits

In addition to IMF, other fat traits, and especially abdominal fat, are important selection criteria in chicken breeding. Abdominal fat (AbF) is under complex genetic control and has medium heritability (h^2^ = 0.62 for AbFW, and 0.24 for AbFP) in one of the founding breeds used here [37]. As the trait is economically important but requires post-slaughter measurement, marker-assisted selection could be a more efficient method for genetic selection. Abdominal fat traits have been a focus of QTL mapping studies of chickens and several chromosomes are involved [4].

Seven genes (*RET*, *NPPB*, *SREBF1*, *COL12A1*, *VPS4B*, *BRSK2*, *FOXC1*) containing or near SNPs associated with abdominal fat traits, identified here, had significant different expression in AbF from cohorts of birds with highest and lowest AbF content. Three of the genes, *RET*, *NPPB* and *SREBF1*, are known to be related to lipid metabolism in other species. *RET* increases lipid accumulation in humans, based on a high-throughput siRNA screen with primary (pre)adipocytes [38]. In the present study, *RET* transcripts were profoundly increased in chickens with high AbFW compared to those in the low group. Even more striking was the 72-fold increased abundance of *NPPB* transcripts in birds with high AbFP. Natriuretic peptide B is implicated in a variety of actions [39,40], including an important role in obesity and insulin resistance [41]. *SREBF1* encodes a transcription factor with roles in adipocyte differentiation and regulation of lipogenesis [42]. Expression of this gene was greatly diminished in the chickens with high AbFP and it is expressed at lower levels in adipose tissue from obese human subjects [43].

Expression of the other genes examined (*COL12A1*, *VPS4B*, *BRSK2*, and *FOXC1*) was significantly lower in chickens with high AbFW or AbFP, though the magnitude of the differential expression was less.

The authors are aware that these traits, measured here at d 93 for their relevance to chicken meat production, reflect cumulative cellular, developmental and metabolic processes, some of which are set in place at much earlier stages. The leads provided by the present GWAS, and general verification by the expression analyses comparing phenotypic extremes, now require a systematic ontogenic analysis from before hatching, where feasible. In the case of adipose tissue, which is non-discernible pre-hatch, attempts to functionally characterize the relevant genes may be possible with preadipocytes, differentiated *in vitro*.

The present approach has used GWAS and mRNA expression analysis to identify loci and genes influencing meat quality traits in chicken. Fine mapping of causal variants in these associated regions and more thorough functional characterization of these genes will be required, using systematic post-GWAS strategies [44,45].

## Conclusions

In summary, the present GWAS has exposed a total of 33 SNPs having significant association with ten meat quality traits (DM_Br_, DM_Th_, IMF_Br_, AbFW, AbFP, meat color L* and b* values, and skin color L*, a* and b* values). Of the 17 genes near the SNPs associated with IMF_Br_, meat L*, b* values and AbF, 14 were differentially expressed in breast muscle or abdominal fat among subsets of chickens with lowest and highest phenotypic values. These results provide new insight into the molecular mechanisms underlying meat quality traits in chickens.

## Methods

### Ethics Statement

The study was conducted in accordance with the Guidelines for Experimental Animals established by the Ministry of Science and Technology (Beijing, China).

### Experimental animals

The Chinese Academy of Agricultural Science (CAAS) chicken F2 resource population was used. The chickens were raised in stair-step cages under the same recommended environmental and nutritional conditions at the conservation farm of the Institute of Animal Sciences (IAS), CAAS. The population was derived from a cross between Beijing-You (BJY) chickens and Cobb broilers (CB, Cobb-Vantress, Inc.). BJY is a slow-growing Chinese indigenous breed, and CB is a commercial fast-growing broiler strain. Six BJY males were each mated to 12 CB females to generate the F1 generation from which six males and 20 females produced the F2 progeny. F1 males were mated to non-related females using artificial insemination. A total of 367 (184 male and 183 female) F2 chickens in five batches, hatched at two-week intervals, were used.

### Phenotypic traits

At 56 days of age, blood was collected from the brachial vein of chickens by venipuncture using citrated syringes during a routine health inspection. At 93 days, chickens were weighed and killed by stunning and exsanguination, 12 h after feed was withheld. After the carcass composition traits were determined, meat quality traits were measured using methods previously described in detail [24,46,47]. The meat quality traits included subcutaneous fat thickness (SFT), AbFW, AbFP, DM_Br_, DM_Th_, IMF_Br_, IMF_Th_, pHu, DL, SF, and the color of muscle and skin, L*, a*, and b*. Samples from the breast muscle and abdominal fat tissues were snap-frozen in liquid nitrogen then held at −80°C until analysis of relative mRNA expression.

### Genotyping and quality control

Genomic DNA (gDNA) was extracted from blood samples using the phenol-chloroform method. Genotyping was performed by DNALandMarks Inc., Saint-Jean-sur-Richelieur, PQ, Canada using Illumina 60 K Chicken SNP Beadchips. Thirty-nine samples were excluded due to sample call rate < 90%. A total of 15,051 SNPs were removed for failing to meet one or more of the following conditions: SNP call rate < 90%, minor allele frequency (MAF) < 3%, Hardy-Weinberg equilibrium (HWE) test p of < 10^-6^ and SNPs with no assigned chromosome or linkage group. After these quality control steps, 42,585 SNPs remained and were distributed among 28 chromosomes and one linkage group (LGE22). The average physical distance between two neighbouring SNPs was approximately 20.4 Kb (Additional file 2: Table S1).

### Statistical analysis

The population structure was assessed by MDS analysis using PLINK 1.07 software [7,48]. Independent SNP markers were obtained on all autosomes using the indep-pairwise option, with a window size of 25 SNPs, a step of five SNPs, and an r^2^ threshold of 0.2. Pairwise identity-by-state (IBS) distances were calculated between all individuals using these independent SNP markers, and MDS components were acquired using the mds-plot option based on the IBS matrix. The relative kinship matrix was also constructed from these independent SNP markers.

The descriptive statistics of the traits were analysed using the MEANS procedure in SAS 8.0 software (SAS Institute Inc., Cary, NC, USA). Some traits deviated from normality, and Box-Cox or Johnson transformations were implemented with Minitab 15 (Minitab Inc., Quality Plaza, PA, USA).

The GWAS analysis for meat quality traits used the GLM and compressed MLM procedures [17] and was performed by Tassel 3.0 software [49] with 42,585 SNPs passing quality control. Both models were performed with the first MDS component as covariates, with batch and sex as fixed effects. In the compressed MLM, relative kinship matrix was a random effect. For AbFW, eviscerated weight (EW) was used as a covariate in both models. The statistical models were,

(I) $$Y_{\mathrm{ijklmn}}={µ}_{i}+C1_{j}+S_{k}+B_{l}+G_{m}+e_{\mathrm{ijklmn}}$$

(II) $$Y_{\mathrm{ijklmn}}={µ}_{i}+C1_{j}+S_{k}+B_{l}+G_{m}+K_{n}+e_{\mathrm{ijklmn}}$$

where, Y_*ijklmn*_ are phenotypic values, μ_*i*_ is the common mean, C1_*j*_ is the effect of the first principal component, S_*k*_ is the effect of sex, B_*l*_ is the effect of batch of hatching (*l* = 1 - 5), G_*m*_ is the effect of the SNP, K_*n*_ is the random effect of the relative kinship matrix, which was constructed by matrix simple matching coefficients based on the independent SNPs, and this step was followed by compression [17], and e_*ijklmn*_ is the random residual.

The p-value thresholds of “LD adjusted” Bonferroni genome-wide significance were calculated based on the estimated number of effective markers and LD blocks [8,50]. The F2 population was estimated to have 16,760 effective SNPs (Additional file 3: Table S2), based on the “solid spine of LD” algorithm with a minimum D′ value of 0.8 calculated by Haploview [51]. The two significant threshold p-values were 5.96×10^-5^ (1/16,760) for suggestive significance and 2.98 × 10^-6^ (0.05/16,760) for genome-wide significance. A Manhattan plot of the p-value results from the GWAS was produced using R 2.13.2 software [52] with the “gap” package [53].

### Quantitative measurements of the expression of candidate genes by Q-PCR

Candidate genes for IMF_Br_, meat color L* and b* in breast muscle, and AbFW and AbFP, exposed by the GWAS, were assessed in the relevant tissues using Q-PCR. Tissue samples from chickens at the extremes of the phenotypic rankings were assembled as High (n = 6) and Low (n = 6) groups (Table 5). Total RNA was isolated from breast muscle and abdominal fat tissue with the RNAsimple Total RNA kit (TIANGEN BIOTECH, Beijing, China). First-strand cDNA was synthesised from 2 μg total RNA using the Reverse Transcription Kit (Promega, Beijing, China). Power SYBR®Green PCR Master Mix (Applied Biosystems, USA) was used to analyse mRNA expression of the selected genes. Quantitative Real-Time PCR was performed with an ABI 7500 Real-time Detection System (Applied Biosystems, USA). The primers (Additional file 4: Table S3) were designed using Primer Premier 5.0 based on chicken sequences. The amplification was performed in a total volume of 20 μl containing 10 μl of 2 × PCR Master Mix, 100 ng cDNA, 0.5 μl of each primer (10 μmol), and 8.0 μl ddH_2_O. To ensure similar PCR efficiencies (close to 100 %) between the target genes and the reference gene (β-actin), the concentrations of primers and cDNA were optimized, if needed. The following PCR conditions were used: 95°C for 10 min, followed by 40 amplification cycles of 95°C for 15s, 60°C for 20 s and 72°C for 32s. To determine fold-changes in gene expression, the comparative CT method was used [54], calculated as 2^-ΔΔCT^. The results are expressed as the mean fold-change in gene expression from triplicate analyses, using the L group samples as the calibrator (assigned an expression level of 1 for each gene).

**Table 5:** Phenotypic values of the traits in the Low and High groups

**Traits**^**1**^	**Groups**^**2**^	
	**Low (n = 6)**	**High (n = 6)**
IMF_Br_ (%)	1.22 ± 0.31	3.95 ± 0.49**
AbFW (g)	3.11 ± 1.32	70.48 ± 12.96**
AbFP (%)	0.21 ± 0.09	2.95 ± 0.63**
Meat color L*	51.53 ± 1.74	62.50 ± 1.14**
Meat color b*	11.07 ± 0.98	21.23 ± 1.34**

^1^*IMF*_*Br*_ intramuscular fat content in breast muscle, *AbFW* abdominal fat weight, *AbFP* percentage of AbFW to eviscerated weight, L* lightness value, b* yellowness value, ^2^High group (n = 6) consisting of samples from chicken with the highest trait values and Low group (n = 6) with the lowest trait values; **p < 0.01.

## Abbreviations

a*: Redness value; AbFP: AbFW as a percentage of eviscerated weight; AbFW: Abdominal fat weight; AGA: aspartylglucosaminidase; b*: Yellowness value; BCL2: B-cell lymphoma 2; BRSK2: BR serine/threonine kinase 2; BUB3: Benzimidazoles 3 homolog (yeast); CAAS: Chinese Academy of Agricultural Science; CLINT1: Clathrin interactor 1; COL1A2: Collagen type I alpha 2; COL12A1: Collagen type XII alpha 1; CV: Coefficient of variation; DL: Drip loss; DM: Dry matter content; DMBr: Dry matter content in breast muscle (% by weight); DMTh: Dry matter content in thigh muscle (% by weight); EW: Eviscerated weight; FAM105A: Family with sequence similarity 105 member A; FOXC1: Forkhead box C1; FTSJ3: FtsJ homolog 3 (E. coli); GGA: Chicken (*Gallus gallus*) chromosome; GDNA: Genomic DNA; GLM: General linear model; GPR10: G-protein coupled receptor 10; GWAS: Genome-wide association studies; HWE: Hardy-Weinberg equilibrium; IAS: Institute of Animal Sciences; IBS: Pairwise identity-by-state; IMFBr: Intramuscular fat content in breast muscle (% by weight); IMFTh: Intramuscular fat content in thigh muscle (% by weight); KIF2A: Kinesin heavy chain member 2A; Kb: Kilobase; KPNA2: Karyopherin alpha 2 (RAG cohort 1 importin alpha 1); L*: Lightness value; LD: Linkage disequilibrium; MAF: Minor allele frequency; MDS: Multidimensional Scaling; MGST1: Microsomal glutathione S-transferase 1; MLM: Mixed linear model; MXD4: Max-interacting transcriptional repressor MAD4; NPPB: Natriuretic peptide B; NTPCR: Nucleoside-triphosphatase cancer-related; PHu: The ultimate pH (at 24h) of breast muscle; PGD: Phosphogluconate dehydrogenase; PrRPR: Prolactin-releasing peptide receptor-like protein; PSMD12: Proteasome (prosome macropain) 26S subunit non-ATPase, 12; PTPN: Tyrosine-protein phosphatase non-receptor type 2; Q-PCR: Real-time quantitative PCR; QTLs: Quantitative trait loci; RET: RET proto-oncogene; SF: Shear force of the pectoral major muscle; SFT: Subcutaneous fat thickness; SNP: Single-nucleotide polymorphism s; SPINK5: Serine peptidase inhibitor Kazal type 5; SREBF1: Sterol regulatory element binding transcription factor 1; ST8SIA5: Alpha-N-acetyl-neuraminide alpha-2,8-sialyltransferase 5; TBC1D24: TBC1 domain family member 24; TYRO3: TYRO3 protein tyrosine kinase; VPS4B: Vacuolar protein sorting-associated protein 4B.

## Competing interests

The authors declare that they have no competing interests.

## Authors’ contributions

YS contributed to the slaughter experiment, genotyped SNPs, performed the Q-PCR experiment and wrote the manuscript. GZ participated in the design of the study, the slaughter experiment and data analysis. RL contributed to the design of the study, the slaughter experiment, data analysis and drafting of the manuscript. MZ participated in the design of the study and the slaughter experiment. YH, DW and LZ participated in the slaughter experiment and genotyped SNPs. PL participated in the feeding and management of chickens and slaughter experiment. JW supervised the study and participated in its design and coordination. All authors read and approved the final manuscript.

## Supplementary Material

Additional file 1: Figure S1 A Manhattan plot showing the association of all SNPs with meat quality traits from the compressed mixed linear model (MLM). SNPs are plotted on the x-axis according to their position on each chromosome against their association with these traits on the y-axis (shown as -log10 p-value). The dashed line indicates genome-wide association (p-value = 1.00 × 10^-4^), and the solid line indicates significance with a p-value threshold of 5.96 × 10^-5^. Click here for file

Additional file 2: Table S1 Basic information for SNP markers on a physical map. Click here for file

Additional file 3: Table S2 LD blocks in the F2 population. Click here for file

Additional file 4: Table S3 Q-PCR primers used in this study. Click here for file
