# Supplementary material for: The identification of 14 new genes for meat quality traits in chicken using a genome-wide association study
Source: BMC Genomics. 2013 Jul 8;14:458. doi: 10.1186/1471-2164-14-458 (PMC3707761; doi:10.1186/1471-2164-14-458)
Supplement: Additional file 1: Figure S1 — A Manhattan plot showing the association of all SNPs with meat quality traits from the compressed mixed linear model (MLM). SNPs are plotted on the x-axis according to their position on each chromosome against their association with these traits on the y-axis (shown as -log10 p-value). The dashed line indicates genome-wide association (p-value = 1.00 × 10^-4^), and the solid line indicates significance with a p-value threshold of 5.96 × 10^-5^. [file 1471-2164-14-458-S1.pdf]

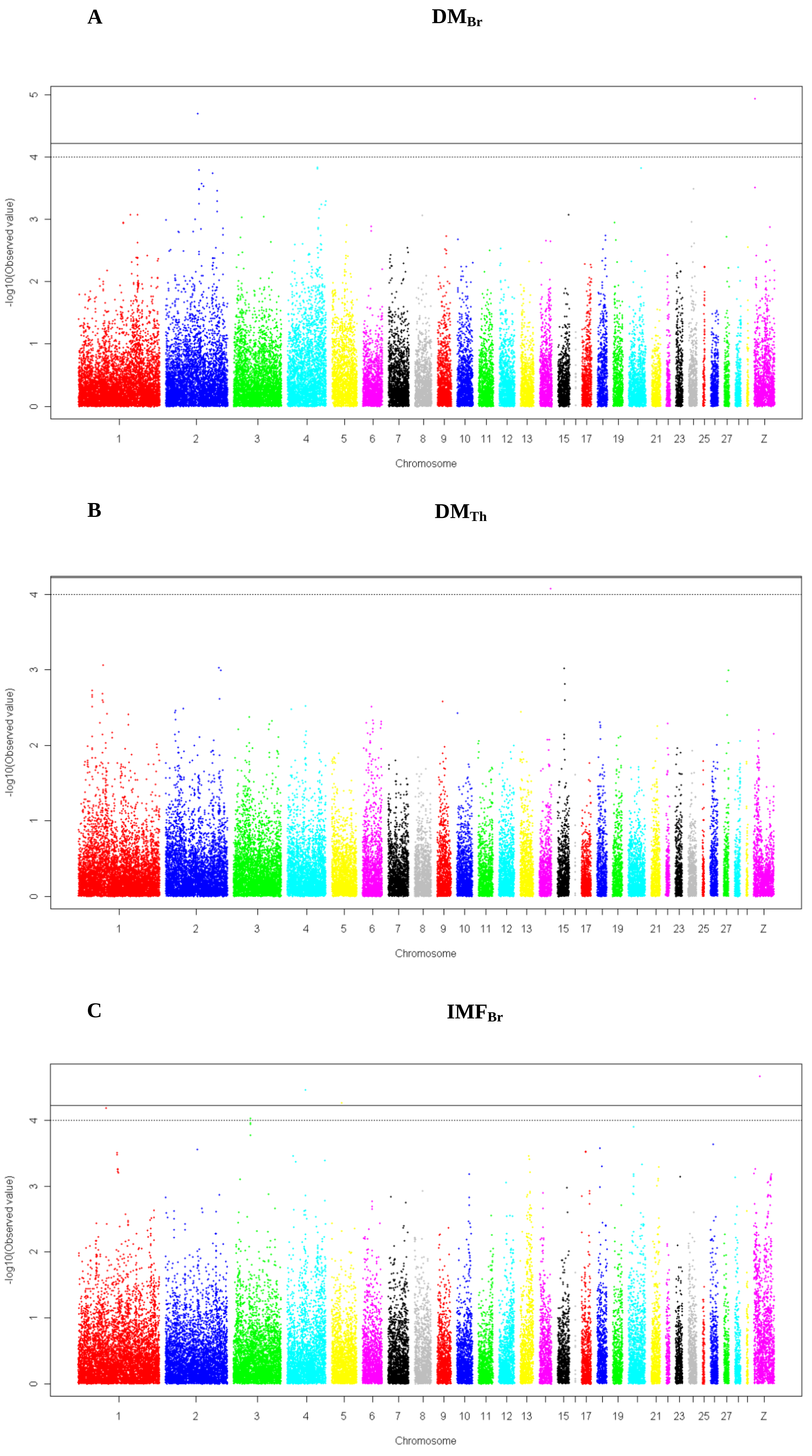
A
DMBr
-log10(Observed value)
Chromosome
1 2 3 4 5 6 7 8 9 10 11 12 13 15 17 19 21 23 25 27 Z
B
DMTh
-log10(Observed value)
Chromosome
1 2 3 4 5 6 7 8 9 10 11 12 13 15 17 19 21 23 25 27 Z
C
IMFBr
-log10(Observed value)
Chromosome
1 2 3 4 5 6 7 8 9 10 11 12 13 15 17 19 21 23 25 27 Z

D **IMF_{Th}**

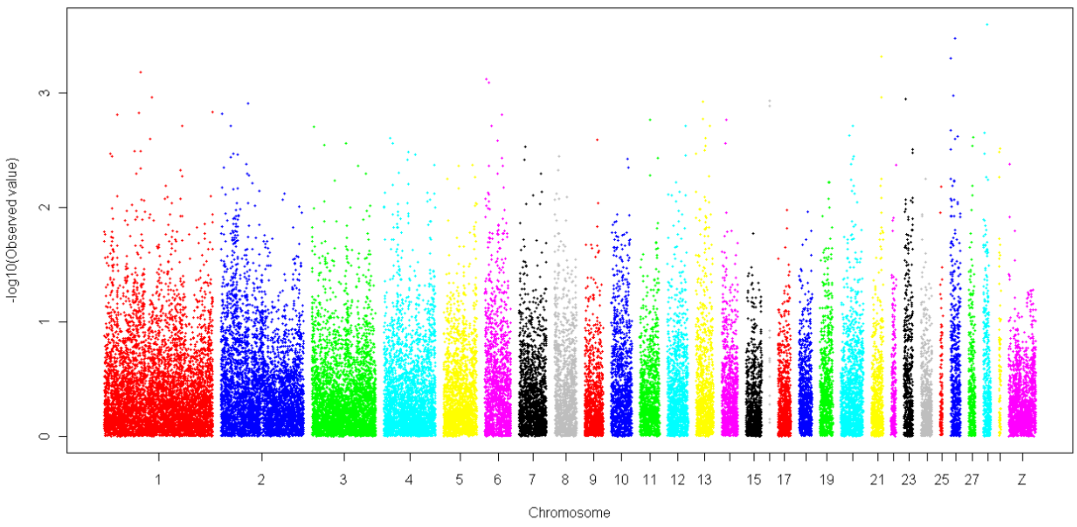

E **SFT**

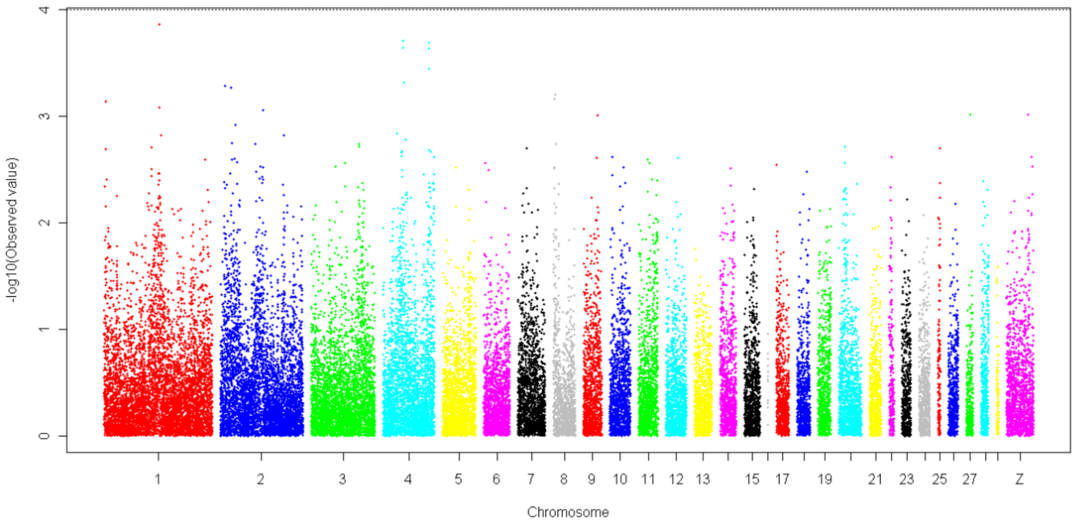

F **pHu**

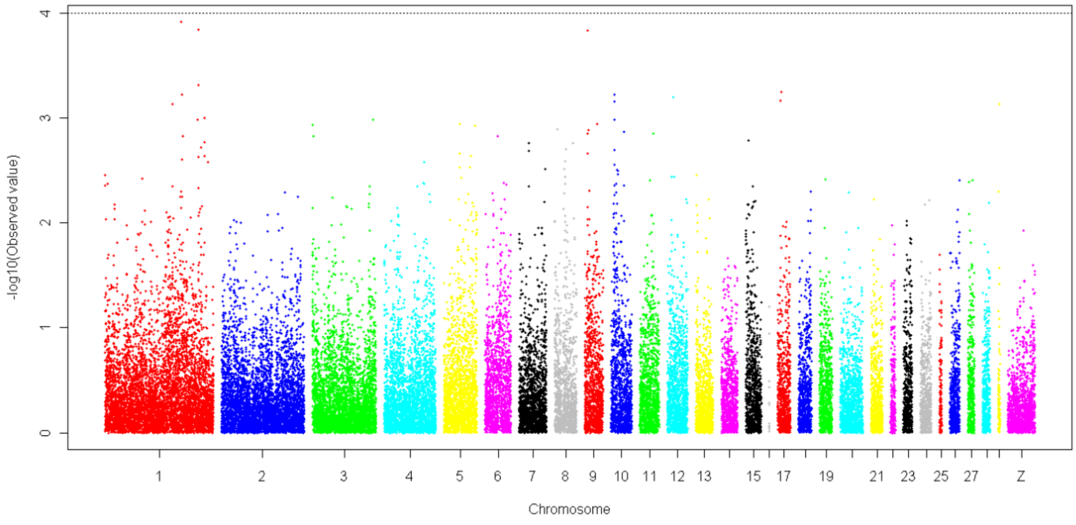

G **DL**

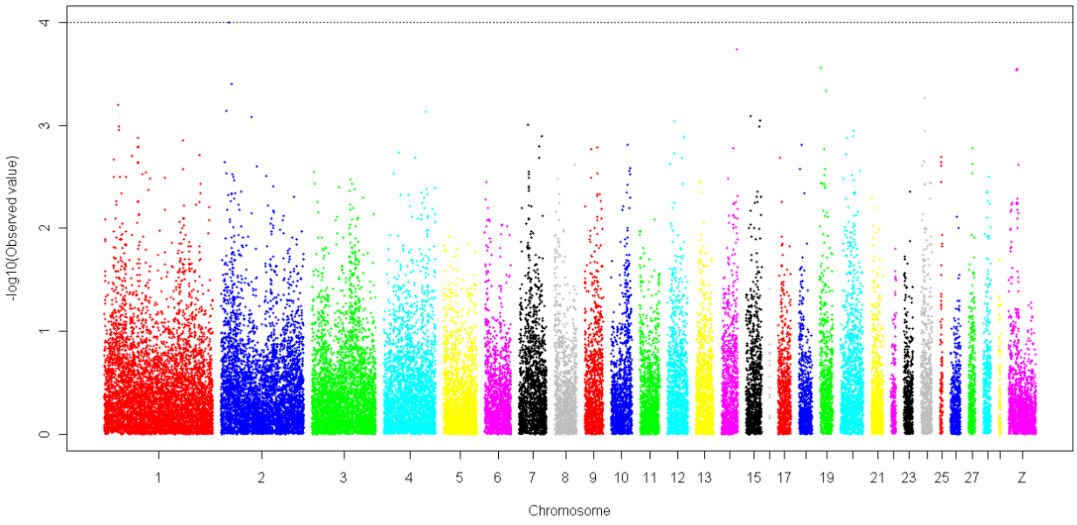

H **SF**

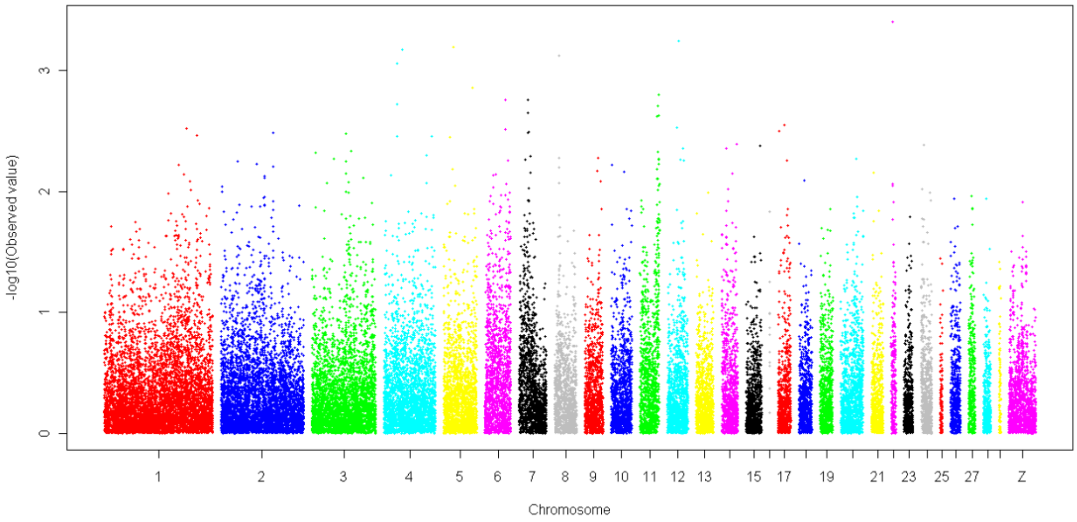

I **Meat color L***

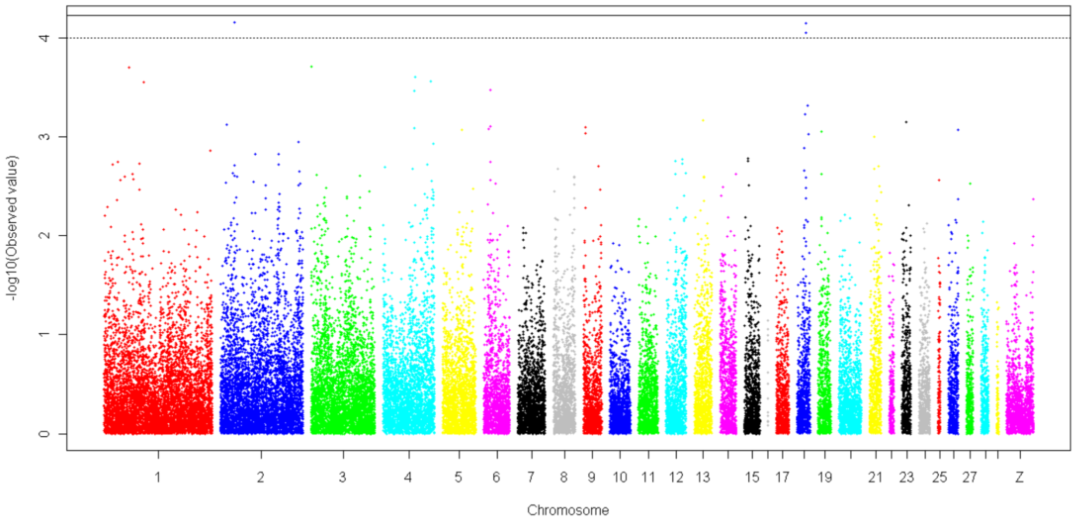

J **Meat color a***

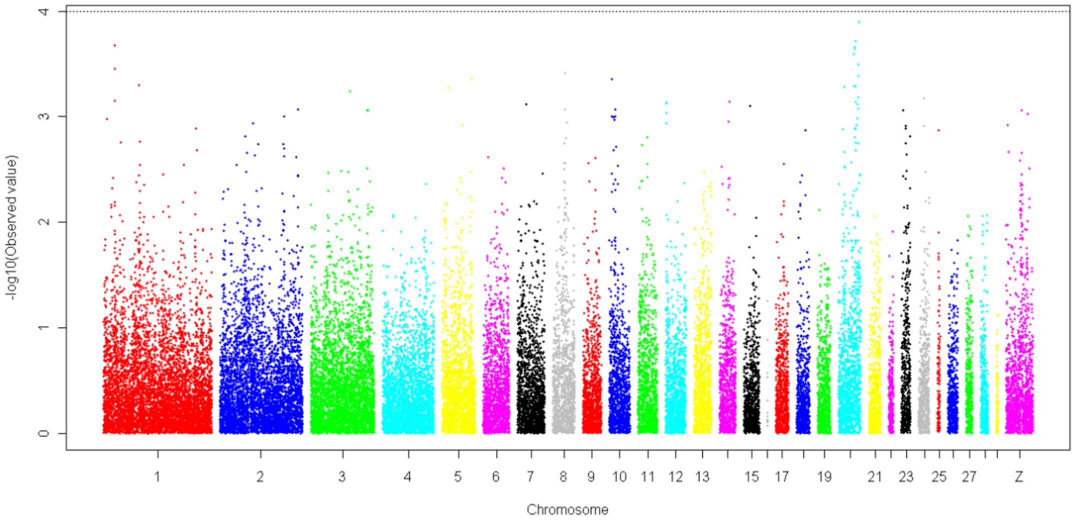

K **Meat color b***

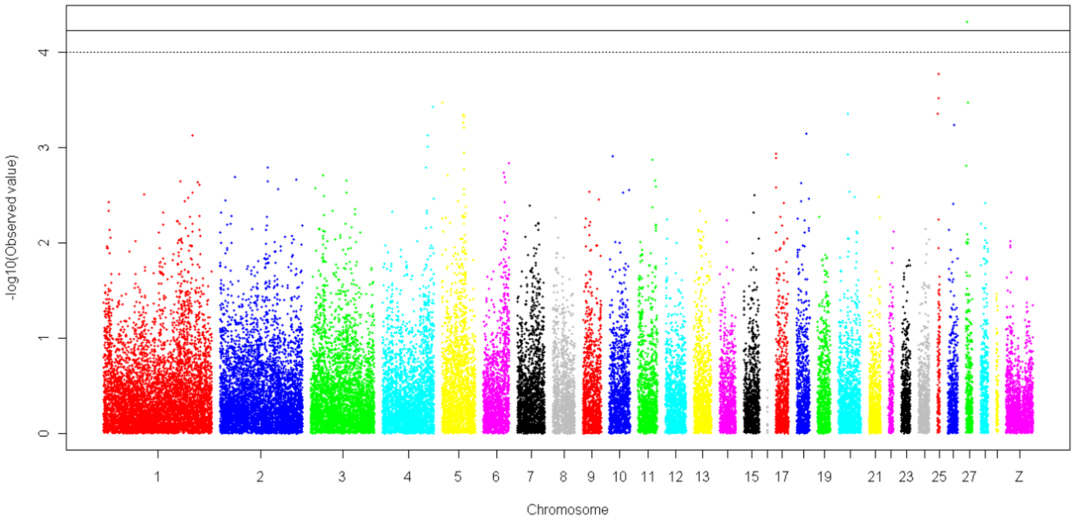

L **Skin color L***

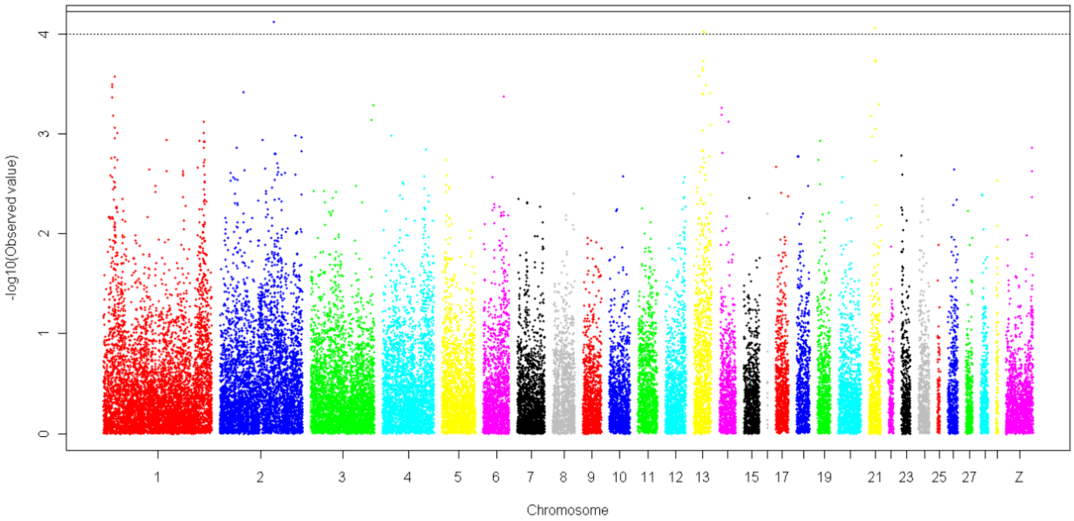

M

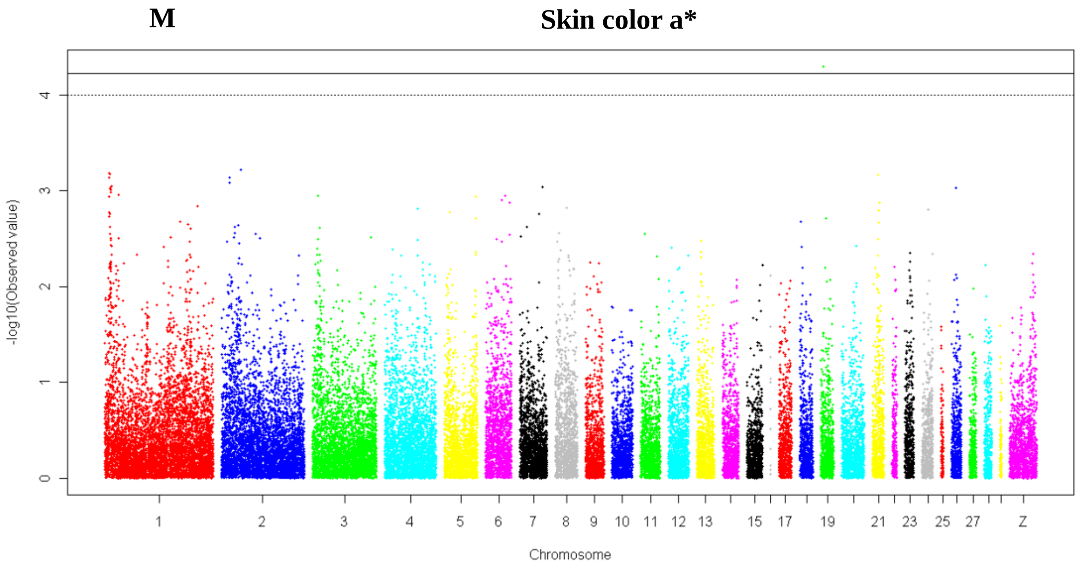
Skin color a*
-log10(Observed value)
0
1
2
3
4
1
2
3
4
5
6
7
8
9
10
11
12
13
15
17
19
21
23
25
27
Z
Chromosome

N

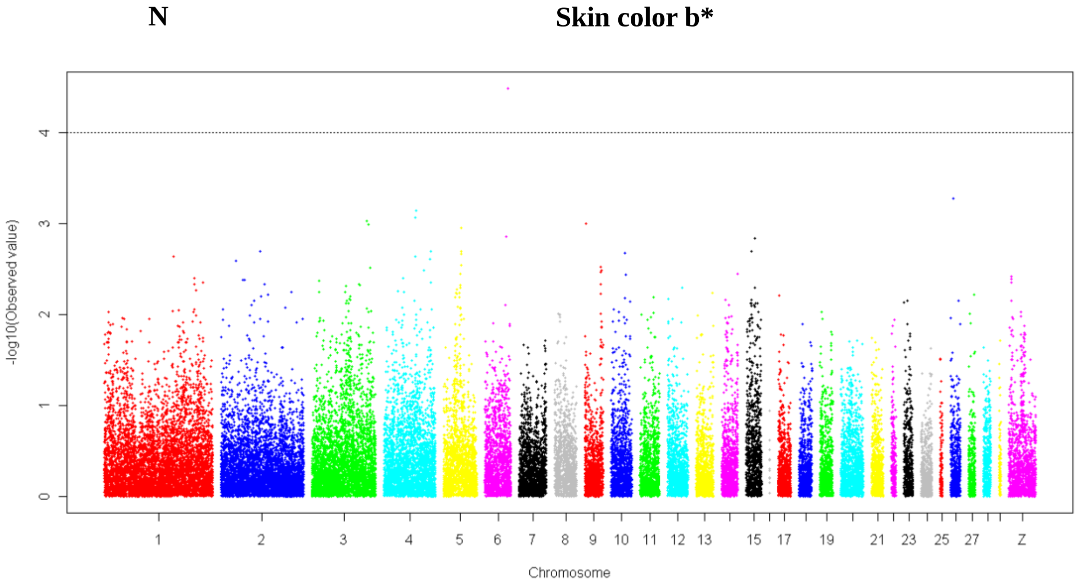
Skin color b*
-log10(Observed value)
0
1
2
3
4
1
2
3
4
5
6
7
8
9
10
11
12
13
15
17
19
21
23
25
27
Z
Chromosome
