# Supplementary material for: The identification of 14 new genes for meat quality traits in chicken using a genome-wide association study
Source: BMC Genomics. 2013 Jul 8;14:458. doi: 10.1186/1471-2164-14-458 (PMC3707761; doi:10.1186/1471-2164-14-458)
Supplement: Additional file 2: Table S1 — Basic information for SNP markers on a physical map. [file 1471-2164-14-458-S2.docx]

**Table S1 Basic information for SNP markers on a physical map**

| **Chromosome** | **Physical distance (Kb)^1^** | **No. of SNPs** | **kb/SNP** | **Chromosome** | **Physical distance (Kb)** | **No. of SNPs** | **kb/SNP** |
| --- | --- | --- | --- | --- | --- | --- | --- |
| 1 | 200994.0 | 6630 | 30.3 | 16 | 433.0 | 12 | 36.1 |
| 2 | 154873.8 | 5061 | 30.6 | 17 | 11182.5 | 785 | 14.2 |
| 3 | 113657.8 | 3915 | 29.0 | 18 | 10925.3 | 784 | 13.9 |
| 4 | 94230.4 | 3150 | 29.9 | 19 | 9939.7 | 774 | 12.8 |
| 5 | 62238.9 | 2027 | 30.7 | 20 | 13986.2 | 1377 | 10.2 |
| 6 | 37400.4 | 1596 | 23.4 | 21 | 6959.6 | 710 | 9.8 |
| 7 | 38384.8 | 1662 | 23.1 | 22 | 3936.6 | 282 | 14.0 |
| 8 | 30671.7 | 1346 | 22.8 | 23 | 6042.2 | 544 | 11.1 |
| 9 | 25554.3 | 1128 | 22.7 | 24 | 6400.1 | 672 | 9.5 |
| 10 | 22556.4 | 1253 | 18.0 | 25 | 2031.8 | 141 | 14.4 |
| 11 | 21928.1 | 1193 | 18.4 | 26 | 5102.4 | 594 | 8.6 |
| 12 | 20536.7 | 1252 | 16.4 | 27 | 4842.0 | 408 | 11.9 |
| 13 | 18911.9 | 1055 | 17.9 | 28 | 4512.0 | 459 | 9.8 |
| 14 | 15819.5 | 1001 | 15.8 | LGE22^2^ | 900.0 | 104 | 8.7 |
| 15 | 12968.2 | 945 | 13.7 | Z | 74602.3 | 1725 | 43.2 |
| Total | 870726.9 | 42585 | 20.4 |  |  |  |  |

^1^The physical length of the chromosome was based on the position of the last marker in the WASHUC2 build (May, 2006); ^2^LGE22 represents linkage group LGE22C19W28_E50C23.
