# Supplementary material for: The identification of 14 new genes for meat quality traits in chicken using a genome-wide association study
Source: BMC Genomics. 2013 Jul 8;14:458. doi: 10.1186/1471-2164-14-458 (PMC3707761; doi:10.1186/1471-2164-14-458)
Supplement: Additional file 3: Table S2 — LD blocks in the F2 population. [file 1471-2164-14-458-S3.docx]

**Table S2 LD blocks in the F2 population**

| **Chromosome** | **Total number**  **of SNPs** | **Total numbers**  **of blocks** | **Total number**  **of interblocks SNPs** | **Total number**  **of blocks + interblocks SNPs^2^** |
| --- | --- | --- | --- | --- |
| 1 | 6630 | 1617 | 1007 | 2624 |
| 2 | 5061 | 1238 | 724 | 1962 |
| 3 | 3915 | 1017 | 624 | 1641 |
| 4 | 3150 | 794 | 468 | 1262 |
| 5 | 2027 | 494 | 317 | 811 |
| 6 | 1596 | 399 | 261 | 660 |
| 7 | 1662 | 409 | 225 | 634 |
| 8 | 1346 | 314 | 196 | 510 |
| 9 | 1128 | 297 | 180 | 477 |
| 10 | 1253 | 321 | 191 | 512 |
| 11 | 1193 | 266 | 164 | 430 |
| 12 | 1252 | 329 | 242 | 571 |
| 13 | 1055 | 259 | 138 | 397 |
| 14 | 1001 | 251 | 139 | 390 |
| 15 | 945 | 234 | 130 | 364 |
| 16 | 12 | 1 | 0 | 1 |
| 17 | 785 | 203 | 129 | 332 |
| 18 | 784 | 220 | 106 | 326 |
| 19 | 774 | 204 | 89 | 293 |
| 20 | 1377 | 354 | 150 | 504 |
| 21 | 710 | 196 | 146 | 342 |
| 22 | 282 | 72 | 40 | 112 |
| 23 | 544 | 146 | 104 | 250 |
| 24 | 672 | 168 | 115 | 283 |
| 25 | 141 | 33 | 18 | 51 |
| 26 | 594 | 151 | 128 | 279 |
| 27 | 408 | 112 | 60 | 172 |
| 28 | 459 | 107 | 63 | 170 |
| LGE22^1^ | 104 | 29 | 22 | 51 |
| Z | 1725 | 296 | 53 | 349 |
| Total | 42585 | 10531 | 6229 | **16760** |

^1^LGE22 represents linkage group LGE22C19W28_E50C23.

^2^ The total number of blocks and interblocks SNPs (16760) is the number of effective SNPs for multiple testing in the Bonferroni correction.
