# Supplementary material for: The identification of 14 new genes for meat quality traits in chicken using a genome-wide association study
Source: BMC Genomics. 2013 Jul 8;14:458. doi: 10.1186/1471-2164-14-458 (PMC3707761; doi:10.1186/1471-2164-14-458)
Supplement: Additional file 4: Table S3 — Q-PCR primers used in this study. [file 1471-2164-14-458-S4.docx]

**Table S4 Q-PCR primers used in this study**

| **Gene name** | **Sequence** | **Production**  **length (bp)** | **GenBank No.** |
| --- | --- | --- | --- |
| *KIF2A* | F 5'- CCGTCCCATTATACACCA -3' | 179 | NM_001039312.2 |
|  | R 5'- GCTCTTCCATTTCCACCA -3' |  |  |
| *AGA* | F 5'- GCTCTGCCCGTAGTCATC -3' | 137 | NM_001006445.1 |
|  | R 5'- CCATCGCACTGGTCAATC -3' |  |  |
| *TYRO3* | F 5'- CTTCACCTCCACTGACATCG -3' | 103 | NM_204627.1 |
|  | R 5'- GCTCCGTAGACTGACTCCAAT -3' |  |  |
| *MGST1* | F 5'- AAATTCCTGCGGACTGAT -3' | 225 | NM_001135550.1 |
|  | R 5'- TGCCCAAGACAAACCTCT -3' |  |  |
| *NTPCR* | F 5'- CAGTTGGTTCTGCCTGTGCT -3' | 187 | NM_001031523.1 |
|  | R 5'- GTGGCTTTCCCTTAGGTATT -3' |  |  |
| *MXD4* | F 5'- GAGCACCGCTACCTCAAA -3' | 175 | NM_001006460.1 |
|  | R 5'- CAGCATCACTGGCACTTC -3' |  |  |
| *COL12A1* | F 5'- CTACGGATTGAGCAGGAACT -3' | 196 | NM_205021.1 |
|  | R 5'- TTGAAGCTGGGACTGAAACA -3' |  |  |
| *RET* | F 5'- GCAGATGCTGTCGTGGAGTT -3' | 202 | NM_205190.1 |
|  | R 5'- CATTTGGGCTGAAGTGGATT -3' |  |  |
| *VPS4B* | F 5'- ACCTCGCTAGTAAGGCAGCAC -3' | 162 | NM_001006378.2 |
|  | R 5'- CCAAGTATTCCGCACATTTCA -3' |  |  |
| *NPPB* | F 5'- TCCCACCAGAACTCACAG -3' | 189 | NM_204925.1 |
|  | R 5'- AACCCATTCCACTCAAGG -3' |  |  |
| *BRSK2* | F 5'- GAGCCAGAACCAGAGCAA -3' | 116 | NM_001199596.1 |
|  | R 5'- TCACGGAAGCAGCCTAAC -3' |  |  |
| *FOXC1* | F 5'- CCCCAAGGACCTGGTGAAG -3' | 173 | NM_205006.1 |
|  | R 5'- GGTTGTGGCGGATGGAGTT -3' |  |  |
| *SREBF1* | F 5'- TGGCAAGGTGAAGCAGGAGA -3' | 92 | NM_204126.1 |
|  | R 5'- GGAAGACAAAGGCACAGAGGG -3' |  |  |
| *COL1A2* | F 5'- GCGGTTTCTACTGGATTGA -3' | 128 | NM_001079714.2 |
|  | R 5'- ATACCAGGTCTTAGTGGGAATA -3' |  |  |
| *PSMD12* | F 5'- GTGGCAGCAGGCACTGAAGAG -3' | 229 | NM_001030706.1 |
|  | R 5'- TGTTGCAGGACTGTCGAGGGA -3' |  |  |
| *KPNA2* | F 5'- TGCTGGGATGTGGTGAACTG -3' | 232 | NM_001006209.1 |
|  | R 5'- AGGCCATGATCTACAACTTGCT -3' |  |  |
| *FTSJ3* | F 5'- TCCAGCAGTTCTTCCACA -3' | 212 | NM_001030925.1 |
|  | R 5'- ATAGCCTTCTGCCTTTGG -3' |  |  |
